# The evolution of image reconstruction for CT—from filtered back projection to artificial intelligence

**DOI:** 10.1007/s00330-018-5810-7

**Published:** 2018-10-30

**Authors:** Martin J. Willemink, Peter B. Noël

**Affiliations:** 1 Department of Radiology, Stanford University School of Medicine, 300 Pasteur Drive, Room M-039, Stanford, CA 94305-5105 USA; 2 Department of Radiology, University Medical Center Utrecht, Utrecht, The Netherlands; 3 Department of Radiology, Perelman School of Medicine, University of Pennsylvania, Philadelphia, PA USA; 4 Department of Diagnostic and Interventional Radiology, Technische Universität München, Munich, Germany

**Keywords:** Tomography, x-ray, Image reconstruction, Artificial intelligence

## Abstract

**Abstract:**

The first CT scanners in the early 1970s already used iterative reconstruction algorithms; however, lack of computational power prevented their clinical use. In fact, it took until 2009 for the first iterative reconstruction algorithms to come commercially available and replace conventional filtered back projection. Since then, this technique has caused a true hype in the field of radiology. Within a few years, all major CT vendors introduced iterative reconstruction algorithms for clinical routine, which evolved rapidly into increasingly advanced reconstruction algorithms. The complexity of algorithms ranges from hybrid-, model-based to fully iterative algorithms. As a result, the number of scientific publications on this topic has skyrocketed over the last decade. But what exactly has this technology brought us so far? And what can we expect from future hardware as well as software developments, such as photon-counting CT and artificial intelligence? This paper will try answer those questions by taking a concise look at the overall evolution of CT image reconstruction and its clinical implementations. Subsequently, we will give a prospect towards future developments in this domain.

**Key Points:**

*• Advanced CT reconstruction methods are indispensable in the current clinical setting.*

*• IR is essential for photon-counting CT, phase-contrast CT, and dark-field CT.*

*• Artificial intelligence will potentially further increase the performance of reconstruction methods*.

Since its introduction in 1972 [1, 2], computed tomography (CT) has evolved into a highly successful and indispensable diagnostic tool. The success story of CT is reflected by the number of annual CT exams, which increased yearly with 6.5% over the last decade resulting in a total of 80 million CT scans in 2015 in the USA [3]. After this first tomographic imaging modality was introduced, its technological developments advanced rapidly. The first clinical CT scan took about 5 min, and image reconstruction took approximately the same time [2]. Despite long reconstruction times, image resolution was poor with only 80 × 80 pixels [2]. Nowadays, rotation speeds are accelerated to approximately a quarter of a second per rotation, and detector coverage, along the patient axis, increased up to 16 cm in high-end systems, allowing for imaging the whole heart in a single heartbeat [4]. Resolution of cross-sectional images increased to 512 × 512 pixels for most clinical applications and to 1024 × 1024 pixels or more for state-of-the-art CT scanners [5, 6].

The increasing number of CT exams, however, has a major drawback. Radiation exposure to society has significantly increased since the introduction of CT imaging, which is especially problematic for younger patients. The combination of growing community awareness about exposure-associated health risks [7] and CT communities’ efforts to tackle them has already led to significant reduction in CT dose. The most important way to reduce CT-radiation exposure is clearly to use this technique only when benefits outweigh the risks as well as costs [8]. However, dose-reduction techniques are necessary in case a CT scan is clinically indicated. Multiple dose-reduction methods were introduced, including tube current modulation [9], organ-specific care [10], beam-shaping filters [11], and most importantly optimization of CT parameters. Essential parameters of every CT protocol include tube current (mA), tube voltage (kV), pitch, voxel size, slice thickness, reconstruction filters, and the number of rotations. It is essential to realize that a different combination of parameters enables significantly different image qualities while delivering the same radiation dose to the patient. For example, the combination of large pixels with a smooth filter can provide diagnostic quality for specific indications, while the same acquisition reconstructed with smaller pixels and a sharper filter would provide non-diagnostic quality through a higher level of noise and artifacts. In the clinical routine, radiation exposure is frequently controlled by adjusting the tube current. When decreasing the tube current, one can observe a proportional increase in image noise. Thus, another dose-reduction technique concerns the proper treatment of image noise and artifacts within the reconstruction of three-dimensional data from raw projection data. Originally, CT images were reconstructed with an iterative method called algebraic reconstruction technique (ART) [12]. Due to lack of computational power, this technique was quickly replaced by simple analytic methods such as filtered back projection (FBP). FBP was the method of choice for decades, until the first iterative reconstruction (IR) technique was clinically introduced in 2009. This caused a true hype in the CT-imaging domain. Within a few years, all major CT vendors introduced IR algorithms for clinical use, which evolved rapidly into increasingly advanced reconstruction algorithms. In this paper, we will take a concise look at the overall evolution of CT image reconstruction and its clinical implementations. Subsequently, we will give a prospect towards future developments in sparse-sampling CT [13], photon-counting CT [14], phase-contrast/dark-field CT [15, 16], and artificial intelligence [17].

## From concept to clinical necessity

**Fig. 1:**
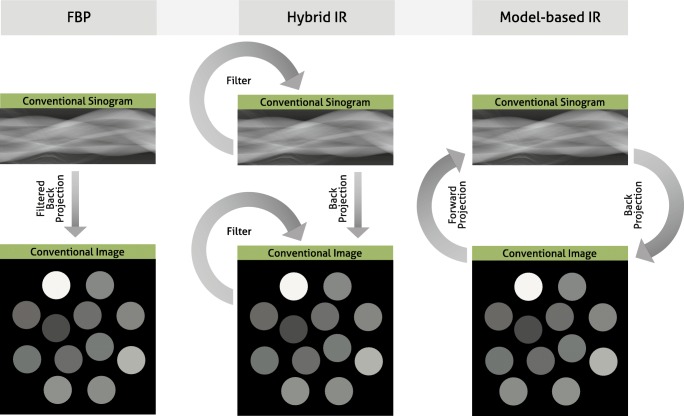
Filtered back projection (FBP), hybrid iterative reconstruction (IR), and model-based IR. With FBP, images are reconstructed from projection data (sinograms) by applying a high-pass filter followed by a backward projection step (left column). In hybrid IR, the projection data is iteratively filtered to reduce artifacts, and after the backward projection step, the image data are iteratively filtered to reduce image noise (middle column). In model-based IR, the projection data are backward projected into the cross-sectional image space. Subsequently, image space data are forward projected to calculate artificial projection data. The artificial projection data are compared to the true projection data to thereupon update the cross-sectional image. In parallel, image noise is removed via a regularization step

In December 1970, Gordon et al presented initial work on ART [18], which is a method belonging to a class of IR algorithms that was initially applied to reconstruct cross-sectional images. However, due to a lack of computation power, ART was not clinically applicable, and a simpler algorithm, namely FBP was standard for decades. With FBP, CT slices are reconstructed from projection data (sinograms) by applying a high-pass filter followed by a backward projection step (Fig. 1A). With the fast progress in CT technology, FBP-based algorithms got improved and extended to keep up with hardware progress, such as Feldkamp et al’s 1984 solution for reconstruction of data from large area detectors [19]. In most circumstances, FBP works well and results in images with high diagnostic quality. However, due to the increasing concerns of exposing (younger) patients with ionizing radiation, more CT scans were being acquired at a lower radiation dose. Unfortunately, this resulted in significantly reduced image quality, because there is a direct proportional relation between image noise and radiation exposure. Also, with the growing prevalence rates of obesity [20], image quality of CT scans reconstructed with FBP deteriorated. With a larger body size, the x-ray photon attenuation increases which leads to less photons reaching the CT detector, finally resulting in significantly reduced image quality. The benefit of FBP is the short reconstruction time, but the major disadvantage is that it inputs raw data into a “black box” where only very limited model and prior information can be applied, for example to properly model image noise when a small number of photons reach the CT detector.

**Fig. 2:**
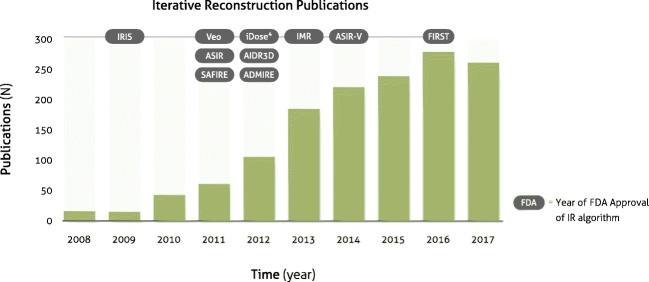
Number of publications on iterative reconstruction for computed tomography. Results based on Pubmed search (“iterative reconstruction” AND (“computed tomography” OR “CT”))

**Fig. 3:**
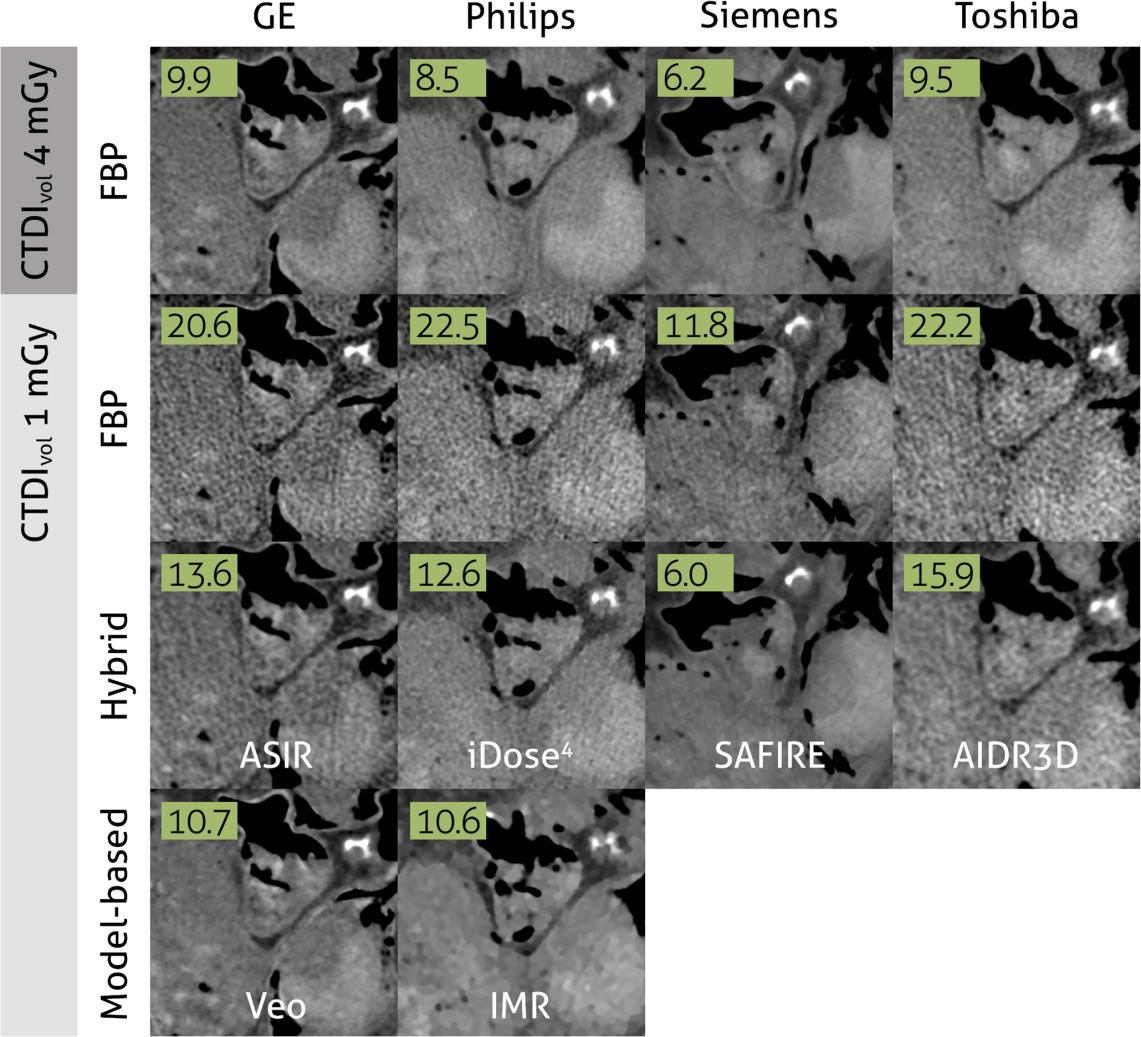
One ex vivo human heart, scanned at 4 mGy and 1 mGy (75% dose-reduction) with high-end CT scanners from four vendors. Images are reconstructed with filtered back projection (FBP), hybrid iterative reconstruction, and model-based iterative reconstruction. Numbers represent noise levels (standard deviations) in air. Images derived from a study published before by Willemink et al [39]

While clinical scanners operated with FBP, the CT research community spent a significant effort into the development of advanced IR algorithms, with the goal to enable low-dose CT with high diagnostic quality. These developments fall loosely into three basic approaches: (i) sinogram-based [21–23], (ii) image domain-based [24–26], and (iii) fully iterative algorithms [18, 27–30]. A parallel progress was an increasing availability of cost-efficient computational tools, such as programmable graphics processing units (GPUs) for accelerated CT reconstruction [31, 32]. This combination of developments has triggered the medical device industry to develop advanced reconstruction algorithms. In 2009, the first IR algorithm called IRIS (iterative reconstruction in image space, Siemens Healthineers) received FDA clearance [33]. This was a simple method that—similar to FBP—only applied a single backward projection step to create a cross-sectional image from raw data. Image noise was iteratively reduced in image space [34]. Within 2 years, four more advanced IR algorithms received FDA clearance: ASIR (adaptive statistical iterative reconstruction, GE Healthcare), SAFIRE (sinogram-affirmed iterative reconstruction, Siemens Healthineers), iDose^4^ (Philips Healthcare), and Veo (GE Healthcare) [35–38]. The first three methods are so-called hybrid IR algorithms. Similar to FBP and IRIS, a single backward projection step is used. However, hybrid IR methods are more advanced since they iteratively filter the raw data to reduce artifacts, and after the backward projection, the image data are iteratively filtered to reduce image noise (Fig. 1B). Veo was the first clinical fully iterative IR algorithm, which was one of the most advanced algorithms so far [39]. In fully IR, raw data are backward projected into the cross-sectional image space. Subsequently, image space data are forward projected to calculate artificial raw data. The forward projection step is a core module of IR algorithms, since it enables the physically correct modulation of the data acquisition process (including system geometry and noise models). The artificial raw data are compared to the true raw data to thereupon update the cross-sectional image. In parallel, image noise is removed via a regularization step (Fig. 1, right column). The process of backward and forward projection is repeated until the difference between true and artificial raw data is minimized. One can imagine that fully IR is computationally more demanding than hybrid IR resulting in longer reconstruction times of fully IR. Due to these long reconstruction times, the vendor decided to develop a different advanced algorithm called ASIR-V (GE Healthcare), which received FDA clearance in 2014. In the meantime, other hybrid and model-based IR algorithms were introduced by other vendors, including AIDR3D (adaptive iterative dose reduction 3D, Canon Healthcare), ADMIRE (advanced modeled iterative reconstruction, Siemens Healthineers), and IMR (iterative model reconstruction, Philips Healthcare) (Table 1). Most recently, in 2016, the model-based IR algorithm FIRST (forward projected model-based iterative reconstruction solution, Canon Healthcare) received FDA-clearance. The introduction of IR for clinical CT imaging resulted in a substantial number of studies evaluating the possibilities of these methods (Fig. 2). Overall, these studies showed improved image quality and diagnostic value with IR compared to FBP. Radiation dose can be reduced with IR by 23 to 76% without compromising on image quality [40]. Some studies compared the different approaches of multiple vendors, and in general, these studies found that radiation dose can be reduced further with model-based IR compared to hybrid IR and FBP [39, 41] (Fig. 3). Multiple studies evaluated the effect of IR on image quality of specific body parts. Relatively low hanging fruit is the CT examination of high-contrast body regions such as the lungs. Due to the low attenuation of x-rays passing through the air in the lungs, and due to the high natural contrast between air and the lung tissue, the radiation dose of chest CT examinations was already relatively low to begin with. In a systematic review of 24 studies, Den Harder et al [42] found that the average radiation dose of 2.6 (1.5–21.8) mSv for chest CT scans reconstructed with FBP could be reduced to 1.4 (0.7–7.8) mSv by applying IR. Similarly, the radiation dose in another high-contrast body region, CT angiography of the heart, could be reduced substantially. With FBP, the average radiation dose of ten coronary CT angiography studies was 4.2 (3.5–5.0) mSv, which could be reduced to 2.2 (1.3–3.1) mSv by using IR, with preserved objective and subjective image quality [43]. Reducing the CT radiation dose of body regions with low contrast such as the abdomen is, however, more problematic [44]. Detectability of low-contrast lesions cannot always be improved with IR at lower radiation doses [45]. However, most studies found that IR does allow for radiation dose reduction of abdominal CT exams without compromising on image quality [46, 47].

**Table 1:** Different iterative reconstruction algorithms from the major vendors

Vendor	Algorithm name	Type of algorithm	Reconstruction speed	Artifact reduction	Noise reduction
GE Healthcare	ASIR (Adaptive Statistical Iterative Reconstruction)	Hybrid	+	+	++
	Veo (MBIR)	Model-based	–	++	+++
	ASIR-V	Hybrid	+	+	++
Philips Healthcare	iDose^4^	Hybrid	+	+	++
	IMR (iterative model reconstruction)	Model-based	–	++	+++
Siemens Healthineers	IRIS (iterative reconstruction in image space)	Image domain	++	–	+
	SAFIRE (sinogram-affirmed iterative reconstruction)	Hybrid	+	+	++
	ADMIRE (advanced modeled iterative reconstruction)	Model-based	–	++	+++
Canon Healthcare	AIDR3D (adaptive iterative dose reduction 3D)	Hybrid	+	+	++
	FIRST (forward projected model-based iterative reconstruction solution)	Model-based	–	++	+++

− minimal; + average; ++ fast/strong; +++ very strong

## Current and future developments

While the number of clinical IR-related publications and the speed of introducing novel clinical algorithms have slowed down, the challenge of reducing radiation exposure remains a topic of high interest. So far, most dose-reduction strategies remained in the domain of decreasing tube current or tube voltage while IR algorithms insure an acceptable diagnostic image quality. A fundamentally different way to reduce radiation exposure is to acquire less projection images, e.g., acquire only every second, fourth, or so projection. This compressed-sensing [48, 49] inspired strategy is widely known as sparse-sampling CT. This approach allows acquiring a reduced number of projections, while the radiation exposure remains high for each individual projection image. The clear benefit of sparse-sampling acquisitions is an improved quality for each individual projection (e.g., increased signal-to-noise ratio) while circumventing the influence of electronic readout noise. Those benefits allow for an additional dose reduction by a factor of two or more when comparing to dose levels achieved with current technology. However, to reconstruct a cross-sectional image from those highly under-sampled data, a fully IR algorithm is imperative. Over the last decade, several investigators have presented IR solutions [50–55], which have the potential to be clinically introduced in the future. Translation into the clinical routine is highly depending on when sparse-sampling capable hardware, e.g., novel x-ray tubes, will become available. However, first evaluations of the clinical potential have been published [56]. One example is the possibility to quantitatively determine bone mineral density (BMD) from the combination of ultra-low-dose sparse-sampling acquisitions and a fully IR algorithm [57].

The integration of advanced prior knowledge into IR algorithms has been a parallel development over the last years. Compared to conventional FBP, IR allows integrating prior knowledge into the reconstruction process. One idea is to utilize previous examination as part of the image formation process. For example, during an oncological follow-up, many patients undergo sequential studies of the same anatomical region. Through the fact that there is shared anatomical information in between the scans, one can utilize this fact in an IR algorithm to significantly improve diagnostic image quality while reducing radiation exposure [58–61]. A different example for prior knowledge is to integrate information concerning orthopedic implants into the reconstruction process. Metal artifact, which can introduce extensive noise and streaks, is caused by implants consisting of materials with high *z* values. However, if the shape and material composition of the implant is known a priori (e.g., from a computer-aided design (CAD) model or a spectral analysis), it has been illustrated that integrating this information into the image formation eliminates artifacts and improves diagnostic image quality [62, 63].

**Fig. 4:**
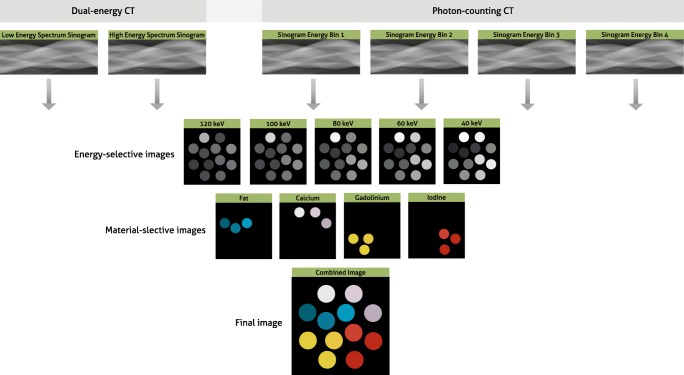
Reconstructions in dual-energy and photon-counting computed tomography. Differentiation of energy levels of x-ray photons allows for the reconstruction of energy-selective images. Material-selective images are reconstructed based on interaction of materials at varying energy levels. Finally, a combined image with different colors per material is reconstructed

Another technology that has found its way into the clinical environment is dual energy CT (DECT). DECT enables material decomposition, which is the quantification of an object composition by exploiting measurements of the material- and energy-dependent x-ray attenuation of various materials using a low- and high-energy spectrum (Fig. 4) [64–66]. This technology has the potential to improve contrast and reduce artifacts as compared to conventional CT. While those advances are becoming clinically available, the issue related to radiation exposure remains, especially for this CT modality. The material decomposition step can significantly intensify image noise when data are acquired with a low radiation exposure. Further, the direct implementation of model-based or fully IR requires several modifications to account for the statistical dependencies between the material-decomposed data. This dependency includes anti-correlated noise, which plays a significant role in the overall image quality in material images. IR-algorithms allow to model anti-correlated noise with a result of significantly improved diagnostic image quality [67–69]. Over the last years, this class of IR specific for DECT has been introduced into the clinical routine. The results can be observed when considering the contrast-to-noise ratio in virtual monoenergetic images (VMI). In theory, a strong increase in noise should be observed towards low VMI (keV) settings and a moderate increase in high VMIs [66, 70]. In DECT scanners with latest IR, one can observe almost no increase in noise for low or high VMI settings [71, 72]. Different DECT acquisition approaches are available including two x-ray tubes with different voltages, one x-ray tube switching between voltages, one x-ray tube with a partly filtered beam, and detector-based spectral separation. Dedicated IR algorithms, accounting for differences in CT design, become necessary for each of these DECT schemes. Further improvements for DECT-specific IR can be expected, for example with the integration of learning algorithms, such as dictionaries [73–75].

An upcoming spectral CT technology, which is gaining clinical interest, is photon-counting CT (PCCT). This unique technology is capable of counting individual x-ray photons while rejecting noise, rather than simply integrating the electrical signal in each pixel. Also, these detectors can perform “color” x-ray detection; they can discriminate the energy of individual photons and divide them into several pre-defined but selectable energy bins, thereby providing a spectral analysis of the transmitted x-ray beam [76–79]. First clinical evaluations illustrated promising performance with respect to quantitative imaging, material specific (K-edge) imaging, high-resolution imaging, and a new level of diagnostic image quality in combination with significant reduction in radiation exposure [80–86]. While first results render the potential benefits of this technology, challenges concerning hardware and software remain. IR plays a central role to overcome those challenges. However, IR algorithms that are used in conventional CT are not optimal for PCCT, because of reasons similar to the statistical dependencies in DECT. For example, PCCT data are more complex than conventional CT data since additional multi-energetic information is present, and additional detector elements can be employed to achieve high spatial resolution images, depending on detector configuration and hardware and software settings. These variations in image acquisition as well as differences in the noise model of PCCT data need to be integrated into the model of the forward projector to fully utilize the power of IR algorithms. One of the reconstruction challenges in PCCT is that the step of material decomposition and image reconstruction are performed separately. This separation implies a loss of information, for which the second step cannot compensate. To adapt IR for this higher level of complexity, image reconstruction and material decomposition can be performed jointly [87]. This can be accomplished by a forward model, which directly connects the (expected) spectral projection measurements and the material-selective images [88–92]. First results illustrated the possibility to overcome challenges related to PCCT, but current IR algorithms are still too computationally intensive, and therefore reconstruction times are too long for clinical use. Further development towards IR solutions with clinical feasible reconstruction times is imperative.

Besides spectral CT, other fundamental CT developments are currently being investigated, namely phase-contrast and dark-field CT. Image contrast in current CT imaging is based on a particle model describing the physical interaction of photoelectric absorption and Compton scattering. Phase-contrast and dark-field CT are based on an electromagnetic wave model, and thus image contrast represents wave-optical interactions such as phase-shift or small-angle scattering. These novel imaging methods make use of these wave optical characteristics of x-rays, by applying for example a grating interferometer to x-ray imaging [93–98]. Compared to conventional CT, additional and complementary information become available. Phase-contrast CT offers significantly higher soft-tissue contrast [99–101], and dark-field CT offers structural information below the spatial resolution of the imaging system [102–104]. When considering a translation, clinical standards, for example with respect to radiation dose and acquisition time, need to be maintained. To ensure those clinical standards, one path is to reconstruct raw data with tailored IR algorithms. Initial investigations have illustrated the high potential of IR algorithms to enhance the image quality in phase-contrast as well as dark-field CT [105–108]. One challenge was the fact that a CT with continuous rotations seems to be not feasible; however, latest developments in fully IR algorithms have enabled the possibility of a continuously rotating gantry [16, 109, 110]. This is a significant step towards clinical translation of phase-contrast and dark-field CT.

Another emerging technique is artificial intelligence (AI). Besides classification of images, detection of objects and playing games [111, 112], AI has gained substantial interest for its potential to improve reconstruction of CT images [17]. AI, and more specifically machine learning, is a group of methods that is able to produce a mapping from raw inputs, such as intensities of individual pixels, to specific outputs, such as classification of a disease [113]. With machine learning, the input is based on hand-engineered features, while unsupervised deep learning is able to learn these features itself directly from data. Multiple research groups are working on applying AI to improve the reconstruction of CT images. One application is image-space-based reconstructions in which convolutional neural networks are trained with low-dose CT images to reconstruct routine-dose CT images [17, 114, 115]. Another approach is to optimize IR algorithms [116]. Generally, IR algorithms are based on manually designed prior functions resulting in low-noise images without loss of structures [117]. Deep learning methods allow for implementing more complex functions, which have the potential to enable lower-dose CT [117–120] and sparse-sampling CT [121]. These AI techniques have the potential to reduce CT radiation doses while speeding up reconstruction times. Also, deep learning can be used to optimize image quality without reducing the radiation dose, e.g., by more advanced DECT monochromatic image reconstruction [122] and metal artifact reduction [123, 124]. These methods are not yet ready for clinical implementation; however, it is expected that AI will play, in the near future, a major role in CT image reconstruction and restoration. We expect that AI will fit in current clinical CT imaging workflow by enhancing current reconstruction methods, for example by significantly accelerating the reconstruction process since application of a trained network can be instantaneously.

In conclusion, IR is a powerful technique that has arrived in clinical practice, and even more exciting advances can be expected from IR in the near future.

## Abbreviations

ADMIRE: Advanced modeled iterative reconstruction
AI: Artificial intelligence
AIDR3D: Adaptive iterative dose reduction 3D
ART: Algebraic reconstruction technique
ASIR: Adaptive statistical iterative reconstruction
BMD: Bone mineral density
CAD: Computer-aided design
CT: Computed tomography
DECT: Dual energy CT
FIRST: Forward projected model-based iterative reconstruction solution
GPU: Graphics processing unit
IMR: Iterative model reconstruction
IR: Iterative reconstruction
IRIS: Iterative reconstruction in image space
PCCT: Photon-counting CT
SAFIRE: Sinogram-affirmed iterative reconstruction
VMI: Virtual monoenergetic images
